# Supplementary material for: Emergence of Nanoscale Drug Carriers through Supramolecular Self-Assembly of RNA with Calixarene
Source: Int J Mol Sci. 2023 Apr 26;24(9):7911. doi: 10.3390/ijms24097911 (PMC10178118; doi:10.3390/ijms24097911)
Supplement: Supplementary file 1 [file ijms-24-07911-s001.zip › ijms-2283987-supplementary.pdf]

Supplementary Information

Emergence of Nanoscale Drug Carriers through Supramolecular Self-Assembly of RNA with Calixarene

[1] Arbuzov Institute of Organic and Physical Chemistry, FRC Kazan Scientific Center of RAS, 8 Arbuzov Str., 420088 Kazan, Russia

[2] Kazan Institute of Biochemistry and Biophysics, FRC Kazan Scientific Center of RAS, 2/31 Lobachevsky Str., 420111 Kazan, Russia

[3] Institute of Fundamental Medicine and Biology, Kazan (Volga Region) Federal University, 18, Kremlyovskaya Str., 420008 Kazan, Russia

* Correspondence: kashapov@iopc.ru

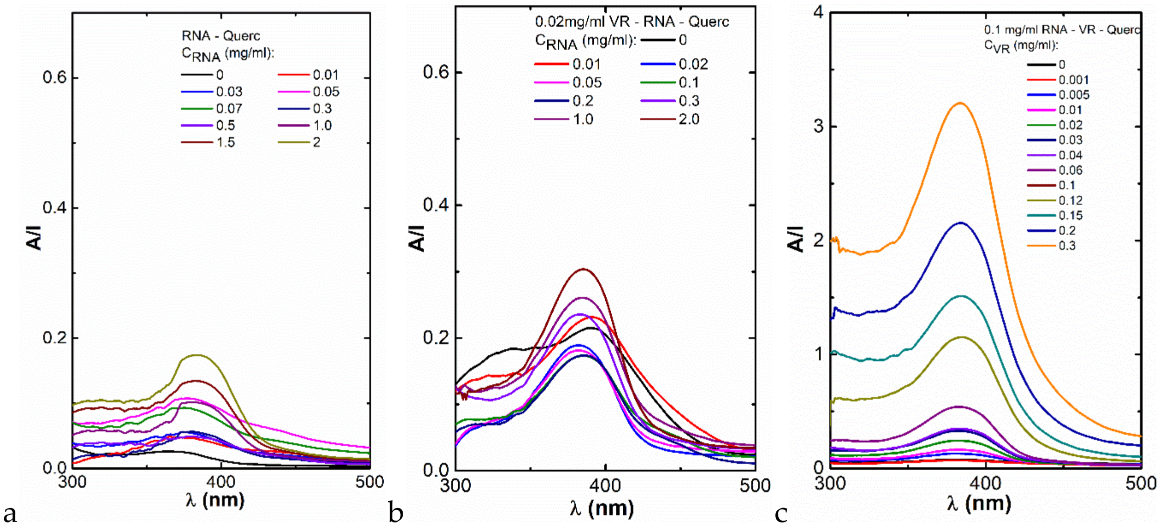

Figure S1. Absorption spectra of quercetin in RNA (a), 0.02 mg/ml VR–RNA (b) and 0.1 mg/ml RNA–VR solutions (c).

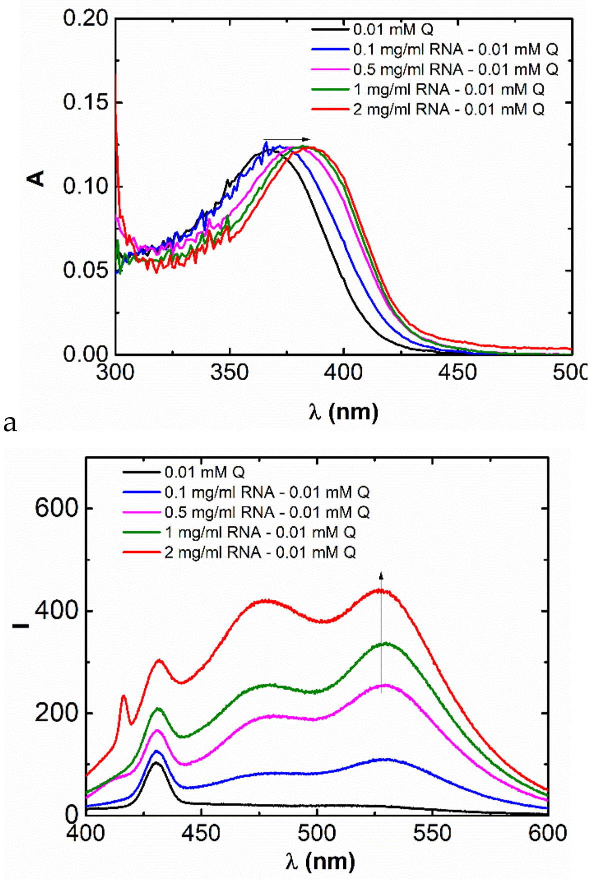

a b

Figure S2. Absorption (a) and fluorescence (b) spectra of 0.01 mM quercetin (ethanol/water=0.5/95.5) in the presence of various amounts of RNA.

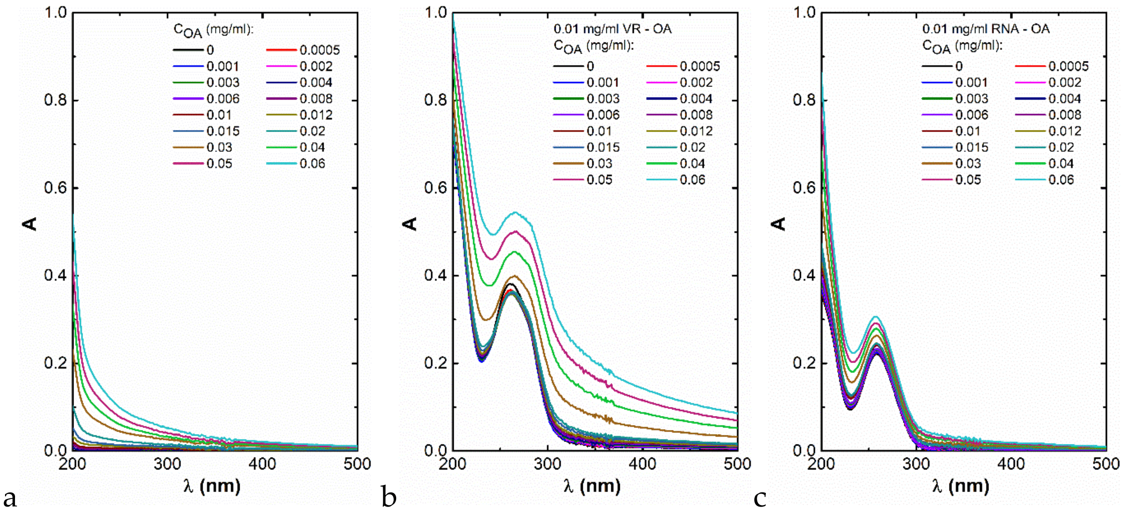

a b c

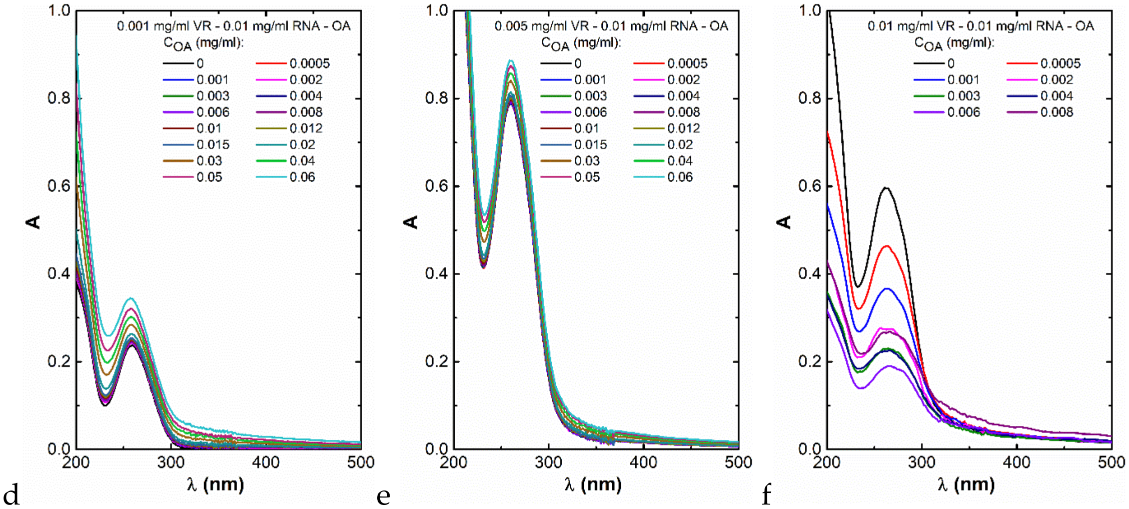

Figure S3. Absorption spectra of oleic acid (OA) in the absence (a) and in the presence of VR (b), RNA (c), VR:RNA 1:10 (d), 1:2 (e) and 1:1 (f) (concentration of ethanol = 0–0.5% v/v).

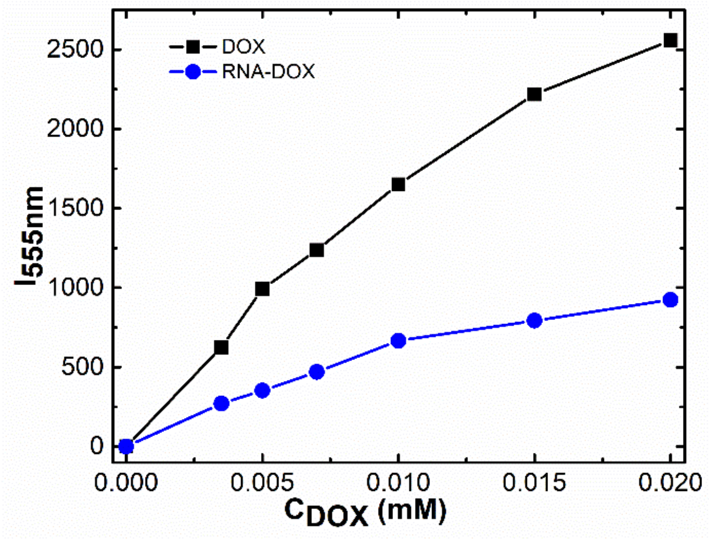

Figure S4. Concentration dependence of DOX fluorescence intensity in the absence and presence of 0.01 mg/ml RNA.

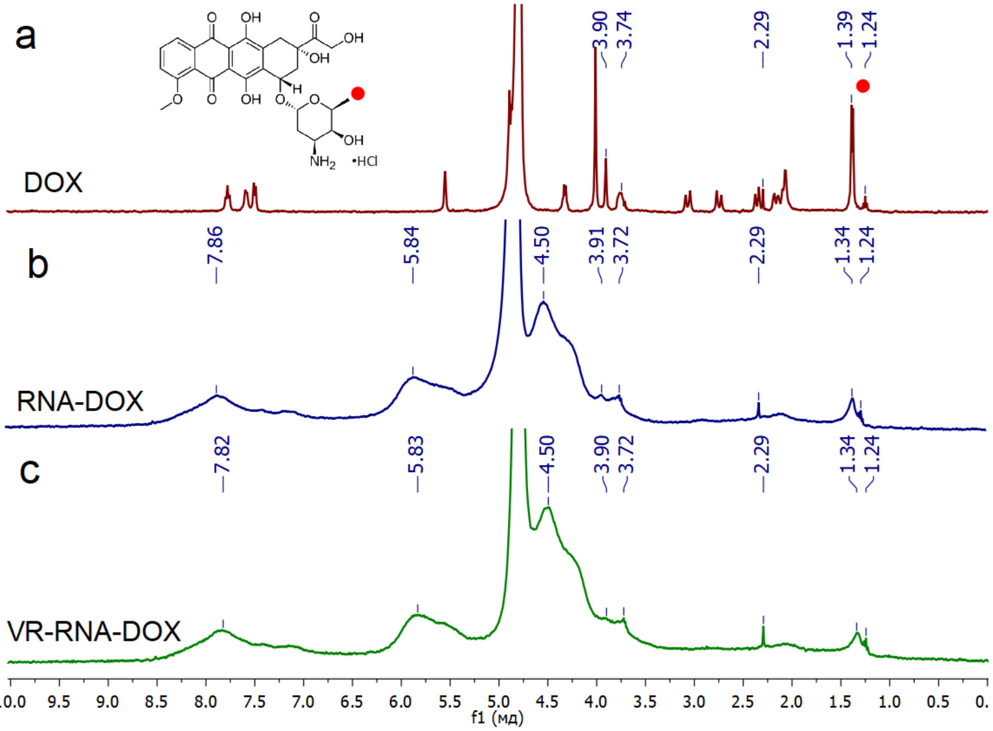

Figure S5. ^{1}H NMR spectra of 1.6 mM DOX (a), 1.6 mM DOX–8 mg/ml RNA (b) and 1.6 mM DOX–8 mg/ml RNA–0.8 mg/ml VR (c) in D_2O.

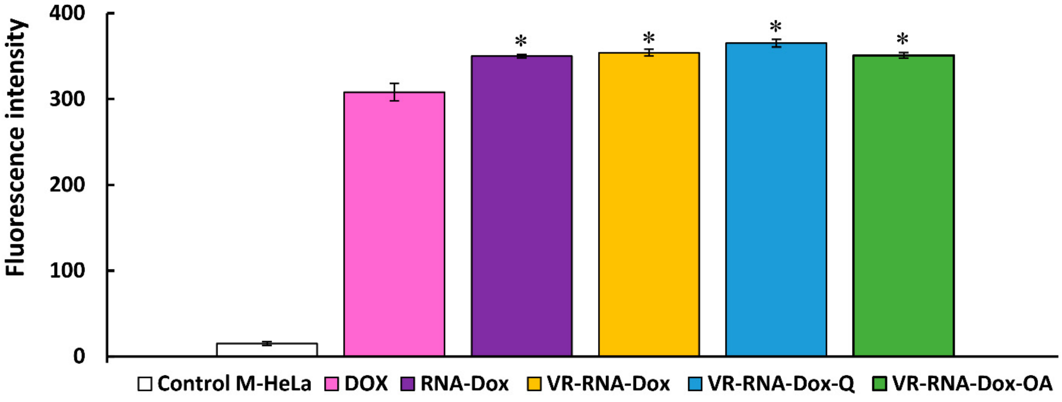

Figure S6. Fluorescence intensity diagram of free and encapsulated DOX in the absence and presence of antioxidants.

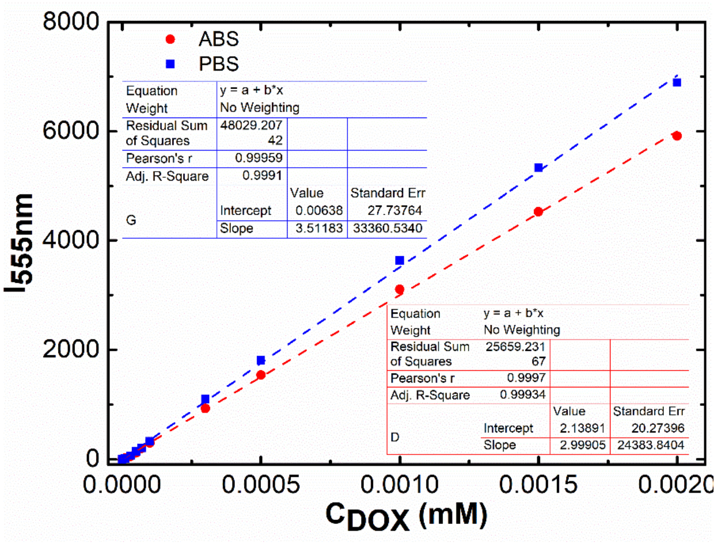

Figure S7. Concentration dependence of emission of DOX at 555 nm in acetate (ABS, pH 5.0) and phosphate buffer solutions (PBS, pH 7.4).
